# The Beginning of the End—The Knell of Sectarianism in Medicine

**Published:** 1902-06

**Authors:** DeLancey Rochester

**Affiliations:** Buffalo, N. Y.


### The Beginning of the End—The Knell of Sectarianism in Medicine.

*Editor Buffalo Medical Journal :*

*Sir.*—An event has recently occurred in medical circles that I think is worthy of more than passing notice. At a general meeting of the medical profession of this city, held in the rooms of the Academy of Medicine last winter, a resolution was presented in which occurred the words "homeopathic physicians." During the discussion of the resolution objection was made by Dr. F. Park Lewis and Dr. A. T. Bull to the use of the word "homeopathic," as indicating that there were any physicians who would allow their field of the scientific investigation and treatment of disease to be limited in such a way as this term implies; that in their opinion it was time to drop such qualifying adjectives; that sectarianism in medicine was really dead and that it was wrong to keep up the semblance of it by the use of such misleading terms; that, so far as they knew, no physician limited himself in his practice in such manner as the word homeopathic implies, and that it was the manly and honest thing to come out openly and say so.

Their words were received with hearty expressions of approval by the members of the profession present at that meeting. As a result of these remarks illustrative of his views, Dr. Lewis was urged by many of his friends to apply for membership in one of the regularly constituted county medical organisations.

At the meeting of the Erie County Medical Association, held May 15, 1902, the name of Dr. Lewis was presented for election to membership.

His application was signed by Dr. A. A. Hubbell, the President of the New York Medical Association and by Dr. Chas. G. Stockton, the chairman of the committee on ethics. It was approved by the committee on membership and Dr. Lewis was elected unanimously.

It seems to the writer that this is a notable occurrence as marking the beginning of the destruction of the barriers that have heretofore separated two large bodies of the medical fraternity. For many years the enlightened members of the medical profession have recognised that the chief barrier was purely academic, that the large body of so-called homeopathic physicians did not limit themselves in the treatment of disease to the use of medicine acting according to the law of "similia similibus," and that on the other hand such remedies were not ignored by the regular profession, although other reasons were found for administering them; that the treatment of disease is becoming less and less a matter of administration of medicine and more and more an art of recognising how by physiologic means to conduct a case from sickness to restoration of health.

Under the circumstances the regular profession should rejoice in the step taken by Dr. Lewis, and should welcome him and all of the brethren who will follow him forward from the ranks of sectarianism to the ranks of the profession of medicine, which recognises no "pathies" or "isms" as applicable to the practice of a true science. In the interest of harmony,

DeLancey Rochester.

Buffalo, N. Y., May 22, 1902.

